# Production of L-Malic Acid by Metabolically Engineered *Aspergillus nidulans* Based on Efficient CRISPR–Cas9 and Cre-*loxP* Systems

**DOI:** 10.3390/jof9070719

**Published:** 2023-06-30

**Authors:** Ziqing Chen, Chi Zhang, Lingling Pei, Qi Qian, Ling Lu

**Affiliations:** 1 Jiangsu Key Laboratory for Microbes and Functional Genomics, Jiangsu Engineering and Technology Research Centre for Microbiology, College of Life Sciences, Nanjing Normal University, Nanjing 210023, China; 2 School of Food Science and Pharmaceutical Engineering, Nanjing Normal University, Nanjing 210023, China

**Keywords:** *Aspergillus nidulans*, CRISPR−Cas9, Cre-*loxP*, metabolic engineering, rTCA, L-malic acid

## Abstract

*Aspergillus nidulans* has been more extensively characterized than other *Aspergillus* species considering its morphology, physiology, metabolic pathways, and genetic regulation. As it has a rapid growth rate accompanied by simple nutritional requirements and a high tolerance to extreme cultural conditions, *A. nidulans* is a promising microbial cell factory to biosynthesize various products in industry. However, it remains unclear for whether it is also a suitable host for synthesizing abundant L-malic acid. In this study, we developed a convenient and efficient double-gene-editing system in *A. nidulans* strain TN02A7 based on the CRISPR–Cas9 and Cre-*loxP* systems. Using this gene-editing system, we made a L-malic acid-producing strain, ZQ07, derived from TN02A7, by deleting or overexpressing five genes (encoding Pyc, pyruvate carboxylase; OahA, oxaloacetate acetylhydrolase; MdhC, malate dehydrogenase; DctA, C4-dicarboxylic acid transporter; and CexA, citric acid transporter). The L-malic acid yield in ZQ07 increased to approximately 9.6 times higher (up to 30.7 g/L titer) than that of the original unedited strain TN02A7, in which the production of L-malic acid was originally very low. The findings in this study not only demonstrate that *A. nidulans* could be used as a potential host for biosynthesizing organic acids, but also provide a highly efficient gene-editing strategy in filamentous fungi.

## 1. Introduction

Over the last couple of decades, via the combination of synthetic biology and computational designs, significant progress has been made in producing valuable compounds using fungi [1]. Based on bioinformatic analyses of the fungal genomes, it has been revealed that fungi display enormous potential to yield significantly more natural products than what has been discovered to date [2]. As a class of common filamentous fungi, species of the genus *Aspergillus,* such as *Aspergillus niger, Aspergillus oryzae,* and *Aspergillus terreus,* have long been regarded as cell factories because of their abilities to survive in varied pH (2–11), a wide temperature range (10–50 °C), and salinity ranges (0–34%), as well as in the other extreme stressful conditions [3]. Therefore, until now, *Aspergillus* species have been increasingly applied to biosynthesize enzymes, food additives, pharmaceuticals, and so on [4].

*A. nidulans* is a well-established model organism for *Aspergilli* as it has a rapid growth and reproduction rate accompanied by a haploid genome and simple nutritional requirements. Because of these traits, fundamental studies in genetics and cell biology and metabolic pathway regulation in *A. nidulans* have been more extensively characterized than those of other *Aspergillus* species [5]. In *A. nidulans*, there are more than 30 biosynthetic gene clusters (BGCs) that have been identified to be related to specific natural products, although most of these gene clusters are silent or expressed at very low levels, and over half of BGCs remain uncharacterized [6]. Recent advances in synthetic biology have enabled the biosynthesis of various secondary metabolites in *A. nidulans,* including benzylpenicillin [7], m-cresol [8], and asperniduglene A1 [9]. The production of primary metabolites in *A. nidulans* is basically limited to those synthesized by certain enzymes including carbohydrate-active enzymes [10], cellulase [11], and lignocellulose-modifying enzymes [12], while other primary metabolites, such as organic acids, have rarely been reported, which somewhat limits the expansion of the application spectrum of *A. nidulans*.

Obtaining high yields of desired products in microbial cell factories often requires gene editing tool-mediated polygenic modification. In the last decade, the CRISPR (clustered regularly interspaced short palindromic repeats)−Cas9 system was developed as a powerful genome-editing tool across various species [13]. Based on different supply modes of Cas9-sgRNA (in vivo or in vitro synthesis) coupled with various types of DNA repair templates, CRISPR−Cas9-based genome editing technologies have been reported in several *Aspergillus* species for gene mutation [14], overexpression [15], point mutation [16], and tag labeling [17]. Tailored CRISPR systems for *A. nidulans* to rapidly and efficiently modify metabolic pathways have not yet been developed, and the traditional homologous recombination method is still the main gene editing strategy for *A. nidulans*; it is time-consuming, laborious, and inefficient for multiple gene editing, which limits the efficient usage of *Aspergillus* resources.

L-malic acid, an important platform chemical, can be produced in many microbial hosts by several metabolic pathways, especially the reductive TCA (rTCA) pathway [18]. The rTCA pathway takes place in the cytosol and involves the carboxylation of pyruvate to form oxaloacetate, and it is catalyzed by pyruvate carboxylase, followed by the reduction of oxaloacetate to malic acid by malate dehydrogenase. The C4-dicarboxylate transporter-mediated efflux of L-malic acid is a crucial step for rapid product accumulation [19]. Enhancing the rTCA pathway and efflux of L-malic acid by a genetic method significantly increased the production of L-malic acid in *A. niger* (201 g/L) [20], *A. oryzae* (165 g/L) [21], *Saccharomyces cerevisiae* (232 g/L) [22], and *Pichia kudriavzevii* (199 g/L) [23]. Based on bioinformatics analysis, *A. nidulans* also harbors all rTCA pathway-related genes and C4-dicarboxylate transporter-encoding genes; whether it is also an efficient host for synthesizing abundant L-malic acid remains unclear.

In this study, a tailored gene editing and Cre-*loxP*-mediated marker recycling system for *A. nidulans* was established. With this system, one gene could be deleted while overexpressing another gene simultaneously by performing only a single transformation. After three rounds of gene-editing manipulations, five genes were edited to strengthen the rTCA cycle for malate accumulation. The metabolically engineered *A. nidulans* was able to produce 30.7 g/L malate, demonstrating that *A. nidulans* is a good potential host for biosynthesizing organic acids.

## 2. Materials and Methods

### 2.1. Strains, Media, and Culture Conditions

The *A. nidulans* strains used in this study are listed in Table 1. The strains were routinely grown on rich medium (YAG or YUU). YAG consisted of 2% glucose, 0.5% yeast extract, 2% agar, and 1 mL/L trace elements [24], and YUU consisted of YAG, 5 mM uridine, and 10 mM uracil. Transformants were screened on riboflavin-amended minimal medium (MMR), which contained 1% glucose, 2% agar, 1 mL/L trace elements, 50 mL/L 20× salt solution, 1.2 M D-sorbitol, and 6.6 µM riboflavin. For the induction of Cre protein expression under the control of the conditional promoter *Pxylp*, we employed a modified minimal medium (MMX) in which 1% xylose (xylanase A gene promoter from *Penicillium chrysogenum*) was used as the sole carbon source [25]. After xylose induction, strains were cultured on YUU or MMPR, which contained MMR and 2.5 µM pyridoxine. The recipe for the liquid medium was identical to that for the related solid medium, but without agar. The strains were cultured at 37 °C for 2 days.

To obtain the appropriate mycelia, 10^7^ fresh spores were inoculated into seed medium, which consisted of 30 g/L glucose, 0.6 g/L KH_2_PO_4_, 2 g/L urea, 0.5 g/L MgSO_4_·7H_2_O, 0.11 g/L ZnSO_4_·7H_2_O, 0.088 g/L FeSO_4_·7H_2_O, and 6.6 µM riboflavin. The strains were cultured in shake flasks at 37 °C and 200 rpm for 24 h. Then, the seeded strains were transferred to the fermentation medium, which consisted of 80 g/L glucose 0.6 g/L KH_2_PO_4_, 0.2 g/L urea 0.5 g/L MgSO_4_·7H2O, 0.11 g/L ZnSO_4_·7H_2_O, 0.088 g/L FeSO_4_·7H_2_O, 6.6 µM riboflavin, 5 mM uridine, 10 mM uracil, and 1 g/L CaCO_3_, to generate L-malic acid. The cultures were incubated at 37 °C and 200 rpm for 120 h. Additional CaCO_3_ (1 g/L) was added to the fermentation medium every day to adjust the pH to 6.5.

### 2.2. Construction of Plasmids

The Cas9-expressing plasmid FM-6 was constructed in a previous study [17]. The plasmid pCre-*loxP*-pyroA (pCZ) was generated as follows. Using a fragment containing two *loxP* sites, the *Pxylp* promoter, *trpC* terminator, and Cre-recombinase gene were generated by PCR with the primer pair psk529-F1 and psk529-R and a plasmid pSK485 as a template [26]. Then, the fragment was cloned into the pEASY-Blunt Zero vector (TransGen Biotech) to generate the plasmid pzero-Cre-*loxP*. The selection marker, the *pyroA* gene, was amplified using the primer pairs 0-cre-loxp-pyro-F and 0-cre-loxp-pyro-R, and subcloned into pzero-Cre-LoxP using seamless cloning technology with Exnase II (ClonExpress^®^ II One Step Cloning Kit, C112), forming pCZ. pCZ01/CZ02 was generated as follows: the *gpdA/tef* promoter was amplified with the primer pairs 0-gpdA/tef-F and 0-gpdA/tef-R from *the A. nidulans* genome and then subcloned the resulting fragment into pCZ to generate pCZ01/pCZ02. To overexpress the target genes *pyc*/*mdhC* with *tef* promoter, the plasmid pCZ03/pCZ04 was generated as follows: *pyc*/*mdhC* was amplified from the *A. nidulans* genome and subcloned into pCZ02, forming pCZ03/pCZ04. For overexpressing *pyc* with *gpdA* promoter, the gene was subcloned into pCZ01, forming pCZ05. Supplementary Table S1 provides a list of primers with annotations.

### 2.3. Gene Editing

Previously established MMEJ-CRISPR system [17] was employed to edit target genes (*oahA*/AN3805, *cexA*/AN0807, *pyc*/AN4462, *mdhC*/AN6499, and *dctA*/AN1472) in *A. nidulans*. In short, more than 4 μg of in vitro synthesized sgRNA and 5 μg of PCR-amplified DNA repair templates were transformed into isolated protoplasts, as described in previous PEG 4000-mediated transformation protocols [24]. sgRNA was synthesized and purified in vitro according to the instructions in a commercially available T7 MEGAscript kit (Life Technologies), and the OD260/OD280 ratio of sgRNA was tested using a spectrophotometer (OneDrop OD-1000 nanodrop) and the value should be around 2. All of the isolates were verified by diagnostic PCR. The sequences of sgRNA and the related PAM used in this study are listed in Table 2.

### 2.4. Western Blotting

To extract the proteins from *A. nidulans* ZQ01, 10^8^ conidia were inoculated into liquid MMPR at 220 rpm and 37 °C for 24 h. The mycelia were collected, frozen in liquid nitrogen, and ground with a mortar and pestle. In general, protein extraction was performed using a previously described alkaline lysis strategy [27]. The membrane was sequentially probed with a 1:5000 dilutions of anti-Cas9 (Roche Applied Science). The blot was developed by enhanced chemiluminescence (ECL, Amersham, UK).

### 2.5. RNA Extraction for qRT–PCR

qRT–PCR analysis was performed after the related strains were grown in YUU at 37 °C and 220 rpm for 24 h. The total RNA of the related strains was extracted using the UNlQ-10 Column TRIzol Total RNA Isolation Kit (Sangon Biotech, B511361-0020, Shanghai, China) following the manufacturer’s instructions. Then, cDNA synthesis was performed with the HiScript II Q RT SuperMix for qPCR Kit (Vazyme, R323-01, Nanjing, China). qRT–PCR was executed using the ABI One-step fast thermocycler (Applied Biosystems, USA) with AceQ Universal SYBR qPCR Master Mix (Vazyme, Q511-02, Nanjing, China). Independent assays were performed with three replicates, and the transcript levels were calculated using the comparative threshold cycle (ΔCT) and normalized against the expression of the *tubA* mRNA level in *A. nidulans*. The 2^−ΔΔCT^ method was used to determine changes in the mRNA expression.

### 2.6. Cre-LoxP System

The Cre-*loxP* system was used for recycling the selection marker [28]. To induce the recombination of two *loxP* sites, fresh spores were streaked on MMX supplemented with 6.6 µM riboflavin and 2.5 µM pyridoxine for 2 days. The resulting isolates that could not be grown in MMR were selected for future experiments. Diagnostic PCR was carried out to confirm elimination of the Cre-*loxP* and *pyroA* cassette.

### 2.7. Quantification of L-Malic Acid

The collected fermentation broth was treated with 6 M HCl, heated at 80 °C for 30 min, and then filtered through a 0.22 μm filter membrane to obtain the supernatant samples. Organic acids were quantified by high-performance liquid chromatography using a standard high-performance liquid chromatography (HPLC) device (Agilent 1100 Series, Agilent Technologies Inc., Santa Clara, CA, USA) equipped with a Rezex ROA organic acid H+(8%) column (300 by 7.8 mm, 8 m; Phenomenex) and a Rezex ROA organic acid H^+^(8%) guard column (50 by 7.8 mm). The samples were analyzed at 35 °C with 0.5 mM H_2_SO_4_ as the mobile phase and a flow of 0.6 mL/min at a wavelength of 210 nm, according to a method described previously.

## 3. Results and Discussion

### 3.1. Expression of Cas9 in A. nidulans

**Figure 1:**
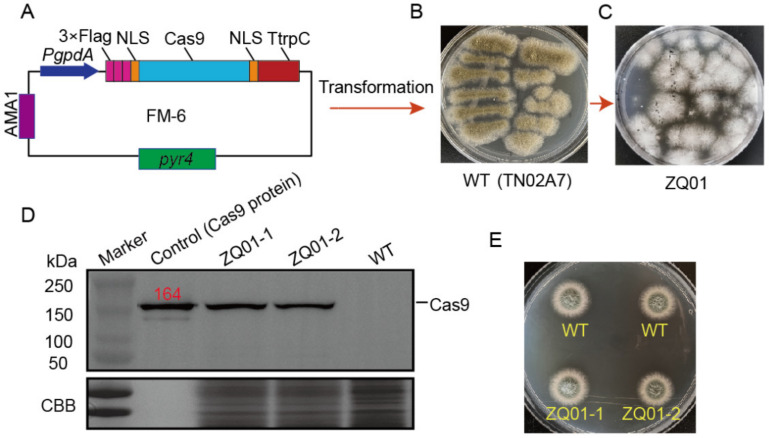
Protein expression of the *cas9* gene with human codon usage bias in *A. nidulans.* (**A**) Schematic illustration of the Cas9-expressing plasmid (FM-6). (**B**) The growth phenotype of WT on YUU. (**C**) The transformation plate generated by introducing plasmid FM-6 into WT. (**D**) Western blotting analysis using an anti-Cas9 antibody indicated that Cas9 was successfully expressed in the ZQ01 strain. Commercial Cas9 and WT were used as the positive control and negative control. (**E**) Two transformants (ZQ01-1/2) of ZQ01 were randomly selected to analyze the growth phenotypes relative to WT on the YUU medium for 2 days.

To modify *A. nidulans* into an excellent chassis cell, efficient and convenient gene editing systems were constructed. Based on the previously identified CRISPR–Cas9 system in *A. fumigatus*, the free plasmid (autonomously maintained in *Aspergillus*) FM-6 (Figure 1A) containing a uridine/uracil auxotrophic marker *pyr4* and a *cas9* gene optimized for codon usage bias in humans was transformed into the parent wild-type strain TN02A7 (WT, Figure 1B). The resulting strain was referred to as ZQ01 (Figure 1C). After purification by the streak plate method, two purified independent transformants were selected and then cultured in liquid medium for 24 h to investigate the Cas9 expression using the Western blotting method. As shown in Figure 1D, using the antibody against Cas9, a specific band at approximately 164 kDa was detected in the ZQ01 and positive control (a commercial Cas9 protein) lanes, but not in the negative control (WT) lane, indicating that the Cas9 protein with human codon usage bias could be expressed at the predicted size in *A. nidulans*. ZQ01 showed a similar colony growth phenotype as its parent wild-type strain on solid medium (Figure 1E), suggesting that the expression of the Cas9 protein had no detectable effect on the colony growth of *A. nidulans*.

### 3.2. Establishment of a CRISPR-Mediated Simultaneous Double-Gene-Editing System in A. nidulans

**Figure 2:**
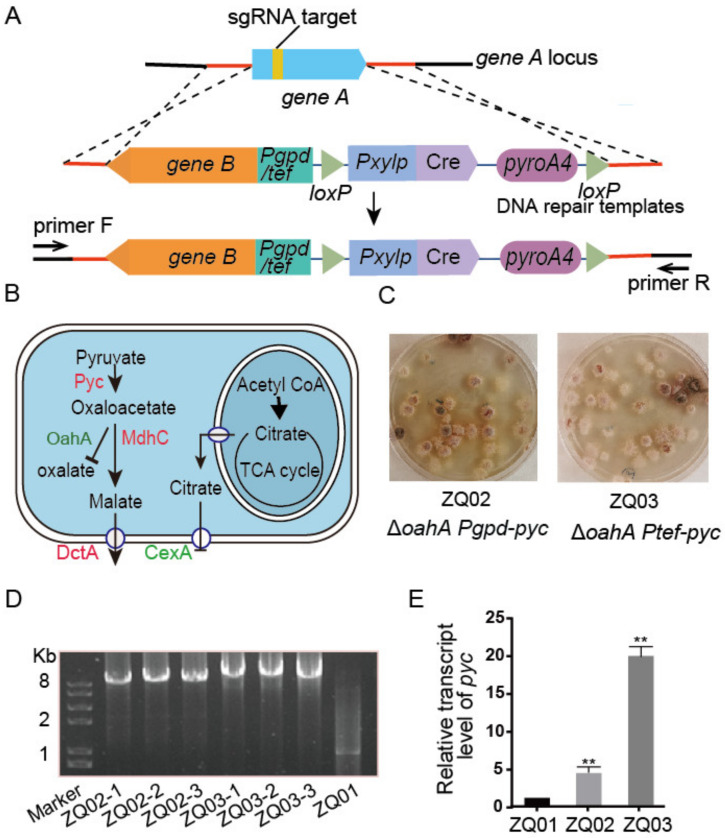
CRISPR-mediated double-gene-editing system in *A. nidulans*. (**A**) Schematic illustration of the CRISPR-mediated double gene editing system. *Pgpd/Ptef* indicates the promoter of the glyceraldehyde-3-phosphate dehydrogenase gene/translation elongation factor EF-1 gene of *A. nidulans*. Primer F and R indicates the diagnostic primer pair. (**B**) Biosynthetic pathway/rTCA pathway of L-malic acid in *A. nidulans*. The red fonts and green fonts represent genes that needed to be overexpressed and deleted, respectively. Pyc, pyruvate carboxylase; OahA, oxaloacetate acetylhydrolase; MdhC, malate dehydrogenase; DctA, C4-dicarboxylic acid transporter; CexA, citric acid transporter. (**C**) Transformation plates generated by introducing repair templates amplified from plasmid pCZ05 and pCZ03 into ZQ01. (**D**) Diagnostic PCR to verify the integration of the corresponding repair template into the *oahA* locus. Three random transformants (ZQ02/ZQ03-1/2/3) were selected. (**E**) The relative transcript levels of *pyc* in related strains growing at 37 °C for 24 h. Values represent the mean ± SD of three replicates. Statistical significance was determined using Student’s *t* test. **, *p* < 0.01.

It is well known that the genetic modification of microorganisms for a high yield of target compounds often relies on multiple rounds of polygenic deletion or/and overexpression. To rapidly achieve the knockout of one gene and overexpression of another gene concurrently by transforming one DNA cassette in *A. nidulans*, we designed a double-gene-editing system based on CRISPR. As shown in Figure 2A, target gene A was designed to be cleaved by the Cas9-sgRNA complex. Subsequently, the resulting nick was repaired using a dedicated repair template/donor DNA harboring target gene B under the control of a constitutive promoter (*Pgpd* or *Ptef*) and the nutritional selection marker *pyroA* packed with the microhomology arms (~30 bp) from gene A. In this system, an in vitro synthesis strategy was used to produce sgRNA, which not only avoids the complex plasmid construction process required for in vivo expression, but also reduces the risk of off-target effects due to the instantaneous production of sgRNA, unlike the continued supply of a gene product by in vivo expression. Nevertheless, the very short (approximately 30 bp) homology arms were enough for the repair template to introduce and integrate extra DNA into the targeted gene region cleaved by the Cas9-sgRNA complex, avoiding a complex assembly of long homology arms using fusion PCR or other molecular cloning techniques. To test the feasibility of our system and develop the capacity of *A. nidulans* in producing typical primary metabolites, genes involved in the rTCA pathway were edited to accumulate L-malic acid. As the initial carbon source substrate in the rTCA pathway, pyruvate can be sequentially converted to oxaloacetate by pyruvate carboxylase (Pyc) and then to malate by malate dehydrogenase (MdhC) (Figure 2B). The metabolic intermediate oxaloacetate can also be hydrolyzed to oxalic acid and acetic acid by oxaloacetate acetylhydrolase (OahA). To simultaneously delete *oahA* and overexpress *pyc*, in vitro synthesized sgRNAs targeting *oahA* and the *pyc* overexpression cassette serving as repair templates, as illustrated in Figure 2A, were cotransformed into the Cas9-expressing strain (ZQ01), generating the ZQ02 (∆*oahA Pgpd-pyc*) and ZQ03 (∆*oahA Ptef-pyc*) strains (Figure 2C). Diagnostic PCR results showed that all of the tested transformants displayed the expected integration of repair template in the *oahA* locus (Figure 2D), indicating that the double-gene-editing system was highly efficient. Although the traditional homologous recombination strategy can also accomplish deletion of a gene and overexpression of another gene simultaneously, the laborious preparatory work for fusing five DNA fragments including two homologous arms (more than 500 bp), a constitutive strong promoter, an open reading frame (ORF) of a target gene, and a selection marker, hinders its popularity. For the CRISPR-mediated double-gene-editing system, the repair template was easily amplified by conventional PCR from a specific plasmid containing promoter, ORF, and selection marker using primer pairs appended with short (approximately 30 bp) homology arms. Importantly, the editing efficiency of this system could reach approximately 100% in the tested transformants.

ZQ03 showed a higher *pyc* expression than ZQ02 and ZQ01 at the mRNA level (Figure 2E), suggesting that the *Ptef* promoter is a better choice for gene overexpression in *A. nidulans*. Well-controlled genetic regulatory elements, especially promoters, are crucial for the construction of engineered strains, but available strong *Aspergillus* promoters are rare. Specific strong promoters for *A. nidulans* under L-malic acid fermentation conditions can be screened using RNA-seq analysis. The overexpression of target genes with specific strong promoters may be effective at increasing the L-malic acid yield.

### 3.3. The Cre-loxP-Mediated Marker Recycling System for Multiple Gene Editing

**Figure 3:**
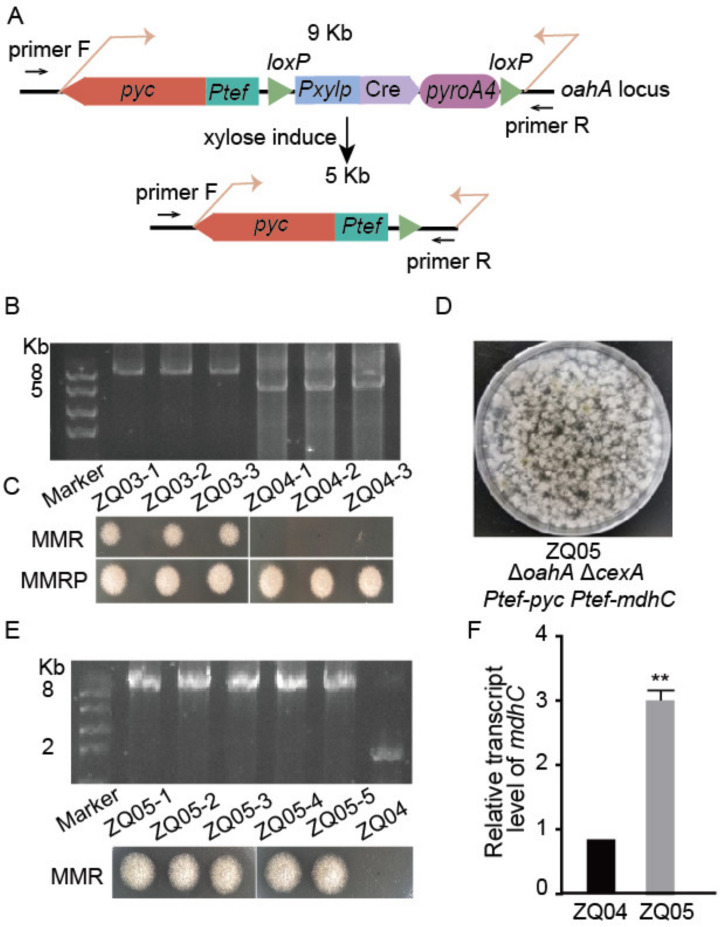
Cre-*loxP*-mediated marker recycling system in *A. nidulans*. (**A**) A simple outline of the Cre-*loxP* system. A *Pxylp*-Cre-*pyroA* cassette is flanked by two key *loxP* sites. After xylose induction, the middle cassette was deleted. Primer F and R indicates the diagnostic primer pair. (**B**) Diagnostic PCR to verify the deletion of the *Pxylp*-Cre-*pyroA* cassette in the original *oahA* locus. Three random isolates were selected. (**C**) The growth phenotypes of ZQ03 and ZQ04 in MMR and MMRP. (**D**) Transformation plates generated by introducing repair template amplified from plasmid pCZ04 into ZQ04. (**E**) Diagnostic PCR to confirm the integration of the related repair template into the *cexA* locus. (**F**) The relative transcript levels of *mdhC* in related strains growing at 37 °C for 24 h. Values represent the mean ± SD of three replicates. Statistical significance was determined using Student’s *t* test. **, *p* < 0.01.

Considering that the lack of selection markers limits the number of rounds of gene editing, the Cre-*loxP*-mediated marker recycling system was employed for editing *oahA* and *pyc*. In this recycling system, Cre (recombinase) can specifically recognize a 34-bp *loxP* site and catalyze reciprocal recombination of the pairs of *loxP* sites, resulting in the removal of the selection marker [28]. As shown in Figure 2A and Figure 3A, the Cre-expressing element under control of the xylose-induced promoter (*Pxylp*) and two *loxP* sites were integrated into the repair template. After induction on solid xylose media, the *Pxylp*-Cre-*pyroA* cassette in the genome of ZQ03 was completely eliminated from the original *oahA* locus, yielding the ZQ04 strain. The deletion of the *Pxylp*-Cre-*pyroA* cassette was verified using PCR (Figure 3B). Pyridoxine-auxotrophy in the ZQ04 strain was confirmed by its failure to grow on media without pyridoxine (Figure 3C), demonstrating that the selection marker *pyroA* could be reused in next-round gene editing. Notably, a recent study by Kohji Yamada et al. also established the CRISPR-Cas9 combined with Cre-*loxP* system used for a marker recycling, through which they identified functional sugar transporters involved in the virulence of *Colletotrichum orbiculare* [29]. Compared with that system, the efficient CRISPR–Cas9 and Cre-*loxP* strategy established in this study not only acted as marker recycling by gene deletion, but could also achieve the inserted gene overexpression by transformation of the same one in *A. nidulans.*

### 3.4. Deleting the Putative Citric Acid Transporter Gene (cexA) and Overexpressing Malate Dehydrogenase Gene (mdhC) in A. nidulans

According to the bioinformatics analysis, the genome of *A. nidulans* contains three malate dehydrogenase-encoding genes (*mdhA*, *mdhB*, and *mdhC*). Among them, the homologous gene (*mdh3*) of *mdhC* in *A. niger* [20] and *A. oryzae* [21] was successfully overexpressed to increase the production of L-malic acid. To further enhance the rTCA pathway, *mdhC* was overexpressed using the *Ptef* promoter. Reportedly, *Aspergillus* tends to accumulate a significant amount of citric acid as a byproduct during fermentation to synthesize malic acid. To block the loss of carbon sources in the form of citric acid, we identified the putative citric acid transporter CexA located in the cell membrane of *A. nidulans* by BLASTP analysis using *A. niger* CexA as a query, and planned to knock out the CexA-encoding gene. Following the double-gene-editing system, the *Ptef*-*mdhC*-*loxP*-*Pxylp*-Cre-*pyroA*-*loxP* cassette was successfully integrated into the *cexA* locus with 100% efficiency in the ZQ04 strain, yielding the ZQ05 strain (Figure 3D,E). The qPCR analysis showed that the *mdhC* expression level was significantly increased in ZQ05 compared with ZQ04 (Figure 3F). The introduction of the Cre-*loxP* system met the requirement for multiple rounds of gene editing.

### 3.5. Overexpressing a Native Putative C4-dicarboxylate Transporter-DctA in A. nidulans

Engineering the C4-dicarboxylate export system is an important strategy for elevating the production of L-malic acid in cell factories. It has been reported that the C4-dicarboxylate transporter C4t318 of *A. oryzae* is competent in the efflux of intracellular L-malic acid in both *A. oryzae* [19] and *A. niger* [20]. The native C4-dicarboxylate transport protein Dct1 in *A. niger* has also been identified as a major L-malic acid transport protein [30]. Using C4T318 or Dct1 as query sequences for BLASTP analysis in the proteome database of *A. nidulans*, DctA (AN1472) showed the highest homology (91% and 95%) to either of these two proteins.

**Figure 4:**
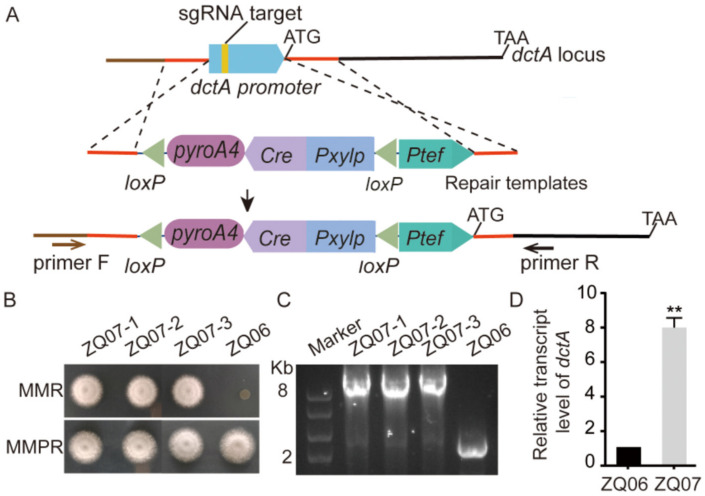
Overexpression of a native putative C4-dicarboxylate transporter in *A. nidulans.* (**A**) Schematic illustration of the CRISPR-mediated *dctA* promoter replacement *in situ*. ZQ07 strain was generated by introducing the sgRNA targeting the native *dctA* promoter region and repair template *loxP*-*Pxylp*-Cre-*pyroA*-*loxP*-*Ptef* cassette into ZQ06. (**B**) The growth phenotypes of ZQ07 and ZQ06 in MMR and MMRP. Three transformants (ZQ07-1/2/3) were selected. (**C**) Diagnostic PCR to confirm the integration of the *loxP*-*Pxylp*-Cre-*pyroA*-*loxP*-*Ptef* cassette in the ZQ07 genome. (**D**) The relative transcript levels of *dctA* in related strains growing at 37 °C for 24 h. Values represent the mean ± SD of three replicates. Statistical significance was determined using Student’s *t* test. **, *p* < 0.01.

To strengthen L-malic acid export in *A. nidulans*, the *dctA* was attempted to be overexpressed by replacing the putatively native *dctA* promoter with Ptef promoter in the pyridoxine-auxotrophic background strain ZQ06 derived from ZQ05 using CRISPR-Cas9 system (Figure 4A), yielding the ZQ07 strain (Figure 4B). Diagnostic PCR verified that all the three tested ZQ07 transformants showed the correct repair template (*loxP*-*Pxylp*-Cre-*pyroA*-*loxP*-*Ptef* cassette) integration in the *dctA* locus.qPCR result confirmed the ZQ07 showed *dctA* overexpression compared to ZQ06 in mRNA level (Figure 4C,D).

### 3.6. A. nidulans Has Good Potential to Produce L-malic Acid

**Figure 5:**
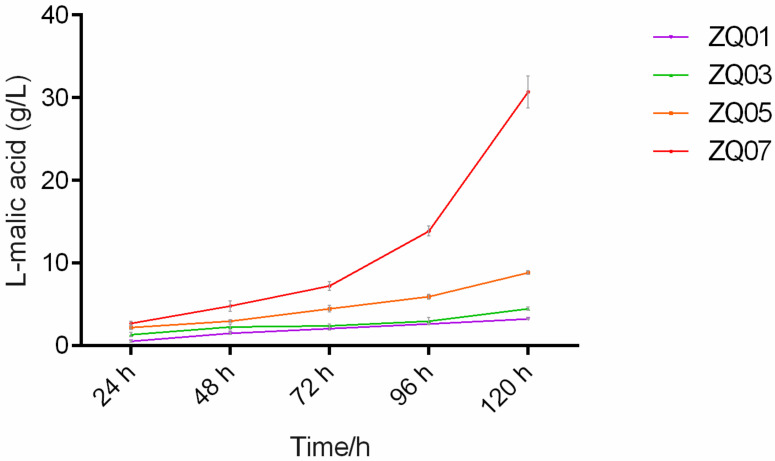
Time course of fermentation of related strains in shake-flask fermentation. The mean values of the L-malic acid titer from three independent experiments at 48 h, 72 h, 96 h, and 120 h are presented.

To measure the L-malic acid production of the genetically engineered *A. nidulans* strains, a two-step-shaken-flask strategy [31] adopted from fermentation parameters in *A. niger* was composed, where step one was the seed culture and step two was cultivation for malic acid production using glucose as the carbon source. As shown in Figure 5, the yield-time curves of L-malic acid in key related strains (ZQ01, ZQ03, ZQ05 and ZQ07) were delineated during fermentation between 24 and 120 h. As a control, the titer of L-malic acid of the ZQ01 strain only reached 3.3 g/L after fermentation for 120 h, suggesting that the TN02A7 background strain was incapable of accumulating L-malic acid at high abundance. After deleting *oahA* and overexpressing *pyc*, the resulting strain, ZQ03, showed slightly elevated L-malic acid production to 4.5 g/L compared to the ZQ01 strain cultured at the time-point of 120 h. Unexpectedly, the four-gene edited strain ZQ05 (∆*oahA* ∆*cexA Ptef-pyc Ptef-mdhC*) displayed a similar level (8.8 g/L) of L-malic acid production as its parent strain, ZQ03. Compared to the low L-malic acid titer of ZQ01, a 9.6-fold increase in the titer up to 30.7 g/L was achieved in the final engineered *A. nidulans* strain ZQ07, which was generated by overexpressing the native putative C4-dicarboxylate transporter DctA in the background of ZQ05, demonstrating that the efflux system of L-malic acid is a key restriction point for L-malic acid accumulation. However, further studying of the correlation between the L-malic acid and *dctA* expression level is needed in our future work since excessive DctA (membrane protein) might be toxic to fungal cells and moderate *dctA* expression may be the most beneficial to L-malic acid yield. It has been reported in yeast *Schizosaccharomyces pombe*, the point mutation F253C/F253A of Mae1 (homolog of DctA and C4t318) is able to improve the transport efficiency of L-malic acid and results in its increased accumulation [23]. Therefore, it suggests that mutating key residues of DctA in *A. nidulans* is also a useful strategy for elevating the yield of L-malic acid in future studies.

From an industrialization perspective, more intensive efforts should be paid to genetic modifications and fermentation optimization for the metabolic engineering *A. nidulans* strain ZQ07 to further enhance its L-malic acid yield. During processes of microbial fermentation for malic acid, the pH value of fermentation needs to be buffered close to 6.5 by the addition of excess CaCO_3_ as a neutralizing agent [17], which probably elicits calcium ion toxicity to *A. nidulans* and damages the acidogenic capacity of cells. Genetic modifications to relieve calcium ion toxicity may be a promising approach for further increasing the L-malic acid production. Optimization of fermentation conditions is an effective strategy for biosynthesis of targeted products, while different *Aspergillus* species may require different optimal fermentation conditions to maximize L-malic acid production. Although current production efficiency of L-malic acid in *A. nidulans* was relatively low compared with that of *A. niger* and *A. oryzae, A. nidulans* still has good potential to elevate production efficiency by optimizing fermentation parameters based on its own characteristics. Notably, at the tested time point of 120 h, the L-malic acid production had not reached a peak value still with sharply increasing tendency. Thus, there might be a good potential to obtain the largest production at the prolonged time point in future studies. To our knowledge, this is the first time to demonstrate *A. nidulans* has the ability to produce relatively abundant L-malic acid.

## 4. Conclusions

In this study, a simultaneous double-gene-editing system for the *A. nidulans* TN02A7 strain was firstly established on the basis of CRISPR–Cas9 and Cre-*loxP* systems. Using this system, three L-malic acid synthesis-related genes (*pyc*, *mdhC* and *dctA*) were overexpressed and two by-product genes (*oahA* and *cexA*) were deleted in the TN02A7 background strain by three rounds of transformation. The final engineered *A. nidulans* strain ZQ07 displayed a 9.6-fold increase in L-malic acid yield up to 30.7 g/L titer under the tested shake-flask fermentation conditions. Our results supported the viewpoint that *A. nidulans* has potential to produce considerable amounts of L-malic acid. Further efforts should be made toward genetic modifications and fermentation optimization to further elevate its L-malic acid yield.

## Figures and Tables

**Table 1:** *A. nidulans* strains used in this study.

Strain	Genotype	Source
TN02A7/WT	*pyrG89; pyroA4; nkuA::argB2; riboB2; veA1*	FGSC
ZQ01/TN02A7^cas9^	*pyrG89; pyroA4; nkuA::argB2; riboB2; veA1*; *pyr4::cas9*	This study
ZQ02/Δ*oahA Pgpd-pyc*	ZQ01, Δ*oahA::Pgpd-pyc-loxP-Pxylp*-Cre*-pyroA-loxP*	This study
ZQ03/Δ*oahA Ptef-pyc*	ZQ01, Δ*oahA::Ptef-pyc-loxP-Pxylp*-Cre*-pyroA-loxP*	This study
ZQ04/Δ*oahA* Δ*pyroA Ptef-pyc*	ZQ01, Δ*oahA::Ptef-pyc,*	This study
ZQ05/Δ*oahA* Δc*exA Ptef-pyc Ptef-mdhC*	ZQ04, Δc*exA::Ptef-mdhC-loxP-Pxylp*-Cre*-pyroA-loxP*	This study
ZQ06/Δ*oahA* Δc*exA* Δ*pyroA Ptef-pyc Ptef-mdhC*	ZQ04, Δc*exA::Ptef-mdhC,*	This study
ZQ07/Δ*oahA* Δc*exA Ptef-pyc Ptef-mdhC Ptef-dctA*	ZQ06, *pryoA::Ptef-dctA*	This study

**Table 2:** The PAM and sgRNA target sequences of *oahA*, *cexA* and *dctA promoter* used in this study.

Target Gene	The Target Regions of sgRNA	PAM Sequence
*oahA*	5′-GGCGGAGTTTGGAGGCAG-3′	5′-CGG-3′
*cexA*	5′-GGACCTAGGATGTGGAAC-3′	5′-TGG-3′
*dctA* promoter	5′- GGGATTCGAAGCTGAGGC-3′	5′-AGG-3′

## Data Availability

All data are publicly available.

## Supplementary Materials

The following supporting information can be downloaded at: https://www.mdpi.com/article/10.3390/jof9070719/s1. Table S1: Primers used in this study.
