# Supplementary material for: Production of L-Malic Acid by Metabolically Engineered Aspergillus nidulans Based on Efficient CRISPR–Cas9 and Cre-loxP Systems
Source: J Fungi (Basel). 2023 Jun 30;9(7):719. doi: 10.3390/jof9070719 (PMC10381526; doi:10.3390/jof9070719)
Supplement: Supplementary file 1 [file jof-09-00719-s001.zip › Table S1.pdf]

Table S1 Primers used in this study

Name	Sequence	Intention
Psk529-F1	CCCTTATTCGAGCTCGGTAC	for amplifying *loxP-Cre-Pxylp-loxP-trpC* fragment
Psk529-R	CCTTCCAAGCTTGCATGCCT	for amplifying *loxP-Cre-Pxylp-loxP-trpC* fragment
O-cre loxp-F	ATACACCGGGCAAAGTTATC	for amplifying *pzero-Cre-loxP* fragment
O-cre loxp-R	GATCTAAGAAGGATTACCTCTAAAC	for amplifying *pzero-Cre-loxP* fragment
O-cre loxp-pyro-F	GGTAATCCTTCTTAGATCTTGGCGGGTAAGTCAGATAA	for amplifying *pyroA* fragment
O-cre loxp-pyro-R	GATAACTTTGCCCGGTGTATCTGACTTGACGCTTTCTCT	for amplifying *pyroA* fragment
0-gpdA-F	AATTAACCCTCACTAAAGGGATCGATAAGCTTGATTAAAGGTTC	for amplifying P*gpdA* fragment
0-gpdA-R	TTTAAACCTGCAGGACTAGTGCATGCGGAGAGACGGACGG	for amplifying P*gpdA* fragment
0-tef-F	TAACCCTCACTAAAGGGGGTGAAGGTTGTGTTATG	for amplifying *Ptef* fragment
0-tef-R	AACCTGCAGGACTAGTGAGACAGCAGAATCACCGCC	for amplifying *Ptef* fragment; as the common R primer for diagnosing *Ptef-dctA*, *Ptef-pyc* and *Ptef-mdhC* cassettes
0-pyc-F	AATTAACCCTCACTAAAGGGCATAACTACTAAGGATGAACCACTG	for amplifying *Pgpd-pyc* fragment; diagnostic primer for *Ptef-pyc* cassette
gpd-pyc-R	CTTTAATCAAGCTTATCGATATGGCTTCCATTGTACCACAC	for amplifying *Pgpd-pyc* fragment
Tef-pyc-R	CATAACACAACCTTCACCATGGCTTCCATTGTACC	for amplifying *Ptef-pyc* fragment
0-mdhC-F	TAACCCTCACTAAAGGGTTACTTTGGTGGGGGACTCTGAG	for amplifying *Ptef-mdhC* fragment; diagnostic primer for *Ptef-mdhC* cassette
tef-mdhC-R	CATAACACAACCTTCACCATGGTCAAGGCTGGTATGTCGAC	for amplifying *Ptef-mdhC* fragment
0-DctA-F	AATTAACCCTCACTAAAGGGCGATACATCCGGAGATGGACATG	for amplifying *dctA* fragment; diagnostic primer for *Ptef-dctA* cassette
Tef-DctA-R	CATAACACAACCTTCACCATGTTCGAAAACGTCCCCTTACC	for amplifying *dctA* fragment
T7-gc-oahA-F	TAATACGACTCACTATAGGGCGGAGTTTGGAGGCAGGTT	for the DNA template of oahA-sgRNA (RNA); for

	TTAGAGCTAGAAATAGCA	deleting *oahA*
Oah-0-gpc-F	GCATACAGCATTCACAAAATGAACACCGCTGCAGAATTAACCCTCACTAAAGGG	for the repair template of constructing *Pgpd-pyc* overexpression
Oah-0-gpc-R	TCAATGCGCTTCCGAGCTTGCACGGCAGCCCGCTTCCTTCCAAGCTTGCATGCC	for the repair template of constructing *Pgpd/Ptef-pyc* overexpression
OahA-yz-F	CTCCACGCTTAACTAAGCATCC	diagnostic primer for Δ*oahA and pyc* overexpression
OahA-yz-R	CCTGGAATACCATCGCGCTTGAG	diagnostic primer for Δ*oahA and pyc* overexpression
T7-gc-cexA-F	TAATACGACTCACTATAGGGTCGCCACAAACACCACGTTTTAGAGCTAGAAATAGCA	for the DNA template of cexA-sgRNA (RNA); for deleting *cexA*
Cex-o-tmc-F	GGAGATTAGTATGGATCAATGCTCATTGGCCAGAATTAACCCTCACTAAAGGG	for the repair template of constructing *Ptef-mdhC* overexpression
Cex-o-tmc-R-1	GTGAAGACCGAATATGGAACCTCGCCGGTGCTTCCTTCCAAGCTTGCATGCC	for the repair template of constructing *Ptef-mdhC* overexpression
Cex-yz-F	GGCTCGATCTTCGTGCCAACGA	diagnostic primer for Δ*cexA* and *mdhC* overexpression
Cex-yz-R	AGGAACCAAGAAAGCACCGGCA	diagnostic primer for Δ*cexA* and *mdhC* overexpression
RT-dctA-F	GACTATCTGCGAGCGGTTAC	qRT-PCR primer
RT-dctA-R	GGTCGATGATGTACACTGCC	qRT-PCR primer
RT-pyc-F	GCCATCATGCAGTTCCAGAAGA	qRT-PCR primer
RT-pyc-R	CCAATCATATAGGCCTCGTCAGC	qRT-PCR primer
RT-mdhC-F	GCTTGCTCTCTACGATATCCGC	qRT-PCR primer
RT-mdhC-R	GTAGCCCTTAACGGTGCTGTTAG	qRT-PCR primer
T7-dct-yw-F：	TAATACGACTCACTATAGGGGATTCGAAGCTGAGGCGGTTTTAGAGCTAGAAATAGCA	for the DNA template of dctA-sgRNA (RNA); for deleting *dctA* promoter
Dct-clp-F:	CCTCACTGGCTTTTACTTCTTCTACCTGCCGCGGCCGCGAATTGGCCCTTC	for the repair template of constructing *Ptef-dctA in situ* overexpression
Dct-yw-R:	AGACTGGGGTAAGGGGACGTTTTCGAACATGGTGAAGGTTGTGTTATGTTTTGTGG	for the repair template of constructing *Ptef-dctA in*

		situ overexpression
Dct-clp-yz-F:	GCAGATCACTGCTCCACTCTCT	diagnostic primer for *dctA in situ* overexpression
Dct-clp-yz-R:	CAGGCCTCCTGTGCTCATGGTC	diagnostic primer for *dctA in situ* overexpression
